# Metabolomics and Network Analyses Reveal Phenylalanine and Tyrosine as Signatures of Anthracycline-Induced Hepatotoxicity

**DOI:** 10.3390/ph16060797

**Published:** 2023-05-26

**Authors:** Peipei Liu, Jing Wu, Xinyue Yu, Linling Guo, Ling Zhao, Tao Ban, Yin Huang

**Affiliations:** 1 Department of Pharmacology, College of Pharmacy, Harbin Medical University, Harbin 150081, China; 2 Key Laboratory of Drug Quality Control and Pharmacovigilance, China Pharmaceutical University, Ministry of Education, Nanjing 210009, China; 3 Department of Pharmaceutical Analysis, School of Pharmacy, China Pharmaceutical University, Nanjing 210009, China; 4 Heilongjiang Academy of Medical Sciences, Harbin 150081, China

**Keywords:** hepatotoxicity, chemotherapy, metabolomics, network

## Abstract

The chemotherapy drug doxorubicin (DOX) is an anthracycline with over 30% incidence of liver injury in breast cancer patients, yet the mechanism of its hepatotoxicity remains unclear. To identify potential biomarkers for anthracycline-induced hepatotoxicity (AIH), we generated clinically-relevant mouse and rat models administered low-dose, long-term DOX. These models exhibited significant liver damage but no decline in cardiac function. Through untargeted metabolic profiling of the liver, we identified 27 differential metabolites in a mouse model and 28 in a rat model. We then constructed a metabolite-metabolite network for each animal model and computationally identified several potential metabolic markers, with particular emphasis on aromatic amino acids, including phenylalanine, tyrosine, and tryptophan. We further performed targeted metabolomics analysis on DOX-treated 4T1 breast cancer mice for external validation. We found significant (*p* < 0.001) reductions in hepatic levels of phenylalanine and tyrosine (but not tryptophan) following DOX treatment, which were strongly correlated with serum aminotransferases (ALT and AST) levels. In summary, the results of our study present compelling evidence supporting the use of phenylalanine and tyrosine as metabolic signatures of AIH.

## 1. Introduction

Anthracyclines are a class of chemotherapy drugs commonly used for the treatment of leukemia, lymphoma, and breast cancer [1]. The most clinically-relevant anthracyclines include doxorubicin (DOX), daunorubicin, epirubicin, and idarubicin [2]. Anthracyclines work by binding topoisomerase-II to break double-stranded DNA, killing both cancer and non-cancer cells [3]. While such drugs have proved effective against cancer, their clinical use is often limited by severe side effects. Two major dose-limiting toxicities associated with anthracyclines are myelosuppression and cardiotoxicity [4,5]. Progress in the therapeutic management of these side effects includes the introduction of cytokines and liposomal formulations [6,7]. However, unlike myelosuppression and cardiotoxicity, other side effects like hepatotoxicity have been less studied. Given the liver’s crucial metabolic functions and the extensive liver metabolism of anthracyclines, the severity of anthracycline-induced hepatotoxicity (AIH) is rising with the trend of higher administration dosages. Clinical data suggests AIH is detected in more than 30% of breast cancer patients [8], so this issue represents a potential clinical challenge and fatal complication.

Treating patients receiving anthracyclines who develop hepatotoxicity requires a solid understanding of the toxicity mechanism. Previous studies have shown significant structural abnormalities (e.g., hepatocyte vacuolation and focal necrosis) and various biochemical alterations (e.g., elevated levels of serum aminotransferases and free radicals) in the livers of animal models treated with DOX [9,10]. The molecular insight into AIH indicates a key role of oxidative stress through the production of reactive oxygen species (ROS), which occurs during the hepatic metabolism of anthracyclines [11]. In addition, mitochondrial dysfunction, inflammation and apoptosis also come into play in AIH [12]. However, metabolic features within the liver under AIH is still largely unknown. Recent developments in metabolomics and network science techniques offer useful tools for a deeper understanding of metabolic changes in drug toxicity [13,14,15]. For example, Timm and colleagues performed untargeted metabolomics analysis on the liver tissue from rats treated with low-dose DOX, whose alanine aminotransferase (ALT) levels were slightly elevated. The analysis uncovered increased and decompensated fatty acid uptake and oxidation, which could lead to oxidative stress and hepatic cellular damage [16]. However, while untargeted metabolomics approaches have been frequently used in AIH studies, these methods may suffer from limitations such as poor reproducibility, limited coverage of the analytical platforms, and bias toward high-abundance molecule detection [17]. Moreover, there is a lack of validation for their findings in tumor-bearing animal models.

This study aims to explore novel and specific metabolic signatures of AIH by utilizing state-of-the-art methodology and clinically relevant animal models. Mouse and rat models of AIH were developed through low-dose, long-term DOX administration, and collected liver tissues underwent untargeted metabolomics analysis using gas chromatography-mass spectrometry (GC-MS) and liquid chromatography-mass spectrometry (LC-MS). Data collected from these models were evaluated using network analysis to uncover potential biomarkers. Findings were further validated in a mouse model of breast cancer with targeted analysis employing liquid chromatography-tandem mass spectrometry (LC-MS/MS).

## 2. Results

### 2.1. Metabolic Alteration in Mouse Liver Caused by AIH

**Figure 1:**
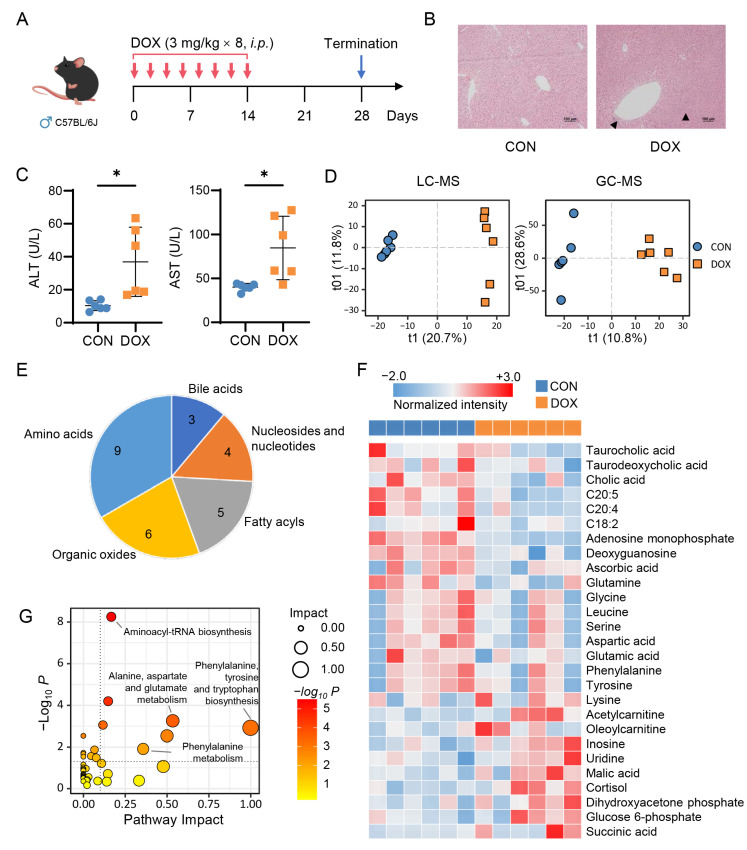
Doxorubicin induces liver damage and metabolic alteration in mice. (**A**) Schematic of a low-dose, long-term doxorubicin (DOX)-induced hepatotoxicity mouse model. (**B**) H&E staining of liver tissues. (**C**) Serum levels of ALT and AST (*n* = 6 per group). Student-t test, * *p* < 0.05. (**D**) OPLS-DA score plot of data obtained from LC-MS and GC-MS analyses (LC-MS, R^2^X = 0.325, R^2^Y = 0.995, Q^2^ = 0.813; GC-MS, R^2^X = 0.394, R^2^Y = 0.959, Q^2^ = 0.328). (**E**) Identified differential metabolites between CON and DOX groups. (**F**) Heatmap of 27 differential metabolites. (**G**) Enriched pathways of 27 differential metabolites.

Previous studies have reported cardiotoxicity as the most severe side effect on the mouse model that is subjected to clinically relevant, low doses of DOX over 2 weeks, followed by 6–8 weeks of observation [18,19]. To create an AIH animal model not suffering heart failure, we performed cardiac echocardiography and biochemical tests at 2, 4, 6, and 8 weeks after the initial administration of DOX in the pilot experiment (Supplementary Figure S1). It was found that mice developed the most severe liver injury at week 4, while heart function was unaffected. Thus, in this study, age and weight-matched male C57BL/6J mice were treated with either sterile saline or DOX (the total dosage of 24 mg/kg) for two weeks and terminated at the 4th week (Figure 1A). By the end of the animal experiment, six mice were left in each group, as two mice treated with DOX died. The histopathological examination showed marked acute cell swelling, accompanied by inflammatory cell infiltration, in the liver of DOX-treated mice (Figure 1B). In addition, elevated serum levels of ALT and aspartate transaminase (AST) indicated remarkable liver damage caused by DOX (Figure 1C).

Next, untargeted metabolomic analysis was performed on liver samples using two platforms, GC-MS and LC-MS. After data preprocessing (see Methods), a total of 4169 features were extracted from GC-MS data and 1498 from LC-MS data (541 from positive ion mode and 957 from negative). QC samples clustered together in the score plots of principal component analysis (PCA), suggesting the stability and repeatability of the analytical systems (Supplementary Figure S2A). Supervised orthogonal projections to latent structures discriminant analysis (OPLS-DA) was applied to characterize the hepatic metabolic patterns of the two groups. As shown in Figure 1D, DOX samples were clearly separated from CON samples in both GC-MS and LC-MS data. A total of 1778 differential features were screened out (Supplementary Figure S2B) between CON and DOX, and 27 differential metabolites with the metabolomics standards initiative (MSI) levels ≤ 2 were finally identified (Figure 1E and Supplementary Table S1). As illustrated in Figure 1F, the levels of amino acids (e.g., phenylalanine, tyrosine, and glutamate) and fatty acids (e.g., linoleic acid [C18:2], eicosapentaenoic acid [C20:5], and arachidonic acid [C20:4]) were down-regulated in the damaged livers comparing with controls, whereas the concentrations of metabolites (e.g., glucose 6-phosphate, malic acid and succinic acid) in glycolysis and tricarboxylic acid (TCA) cycle were up-regulated. We further performed pathway enrichment analysis using MetaboAnalyst 5.0 (https://www.metaboanalyst.ca, 10 February 2023) to identify Kyoto Encyclopedia of Genes and Genomes (KEGG) pathways significantly overrepresented in the 27 differential metabolites (Figure 1G). The data supported that amino acid metabolism was significantly altered following DOX exposure.

### 2.2. Metabolic Alteration in Rat Liver Caused by AIH

**Figure 2:**
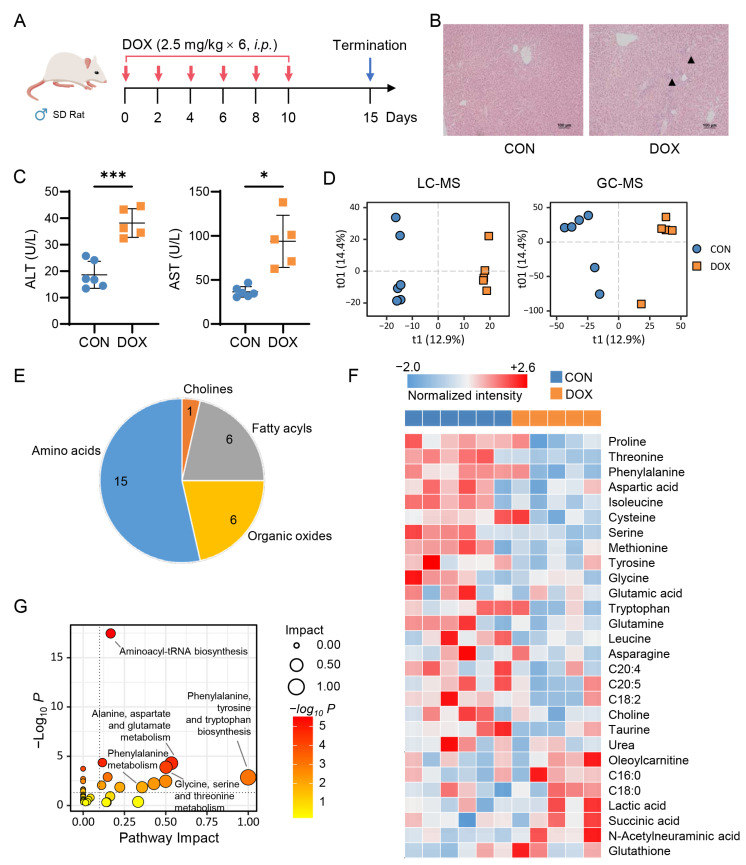
Doxorubicin induces liver damage and metabolic alteration in rats. (**A**) Schematic of a low-dose, long-term doxorubicin (DOX)-induced hepatotoxicity rat model. (**B**) H&E staining of liver tissues. (**C**) Serum levels of ALT and AST (CON, *n* = 6; DOX, *n* = 5). Student-t test, * *p* < 0.05; *** *p* < 0.001. (**D**) OPLS-DA score plot of data obtained from LC-MS and GC-MS analyses (LC-MS, R^2^X = 0.273, R^2^Y = 0.998, Q^2^ = 0.423; GC-MS, R^2^X = 0.478, R^2^Y = 0.918, Q^2^ = 0.398). (**E**) Identified differential metabolites between CON and DOX groups. (**F**) Heatmap of 28 differential metabolites. (**G**) Enriched pathways of 28 differential metabolites.

To further understand the alterations, another AIH animal model was built by treating male Sprague-Dawley (SD) rats with long-term, low-dose of DOX (Figure 2A). Three rats died after DOX administration, leaving five that exhibited significant liver injury, including histopathological changes (Figure 2B) and elevated levels of serum ALT and AST (Figure 2C). Metabolomics analyses of liver samples showed a clear separation between groups (Figure 2D) and a well-clustered set of QC samples (Supplementary Figure S3A). A total of 3596 differential features were screened from the livers of the two groups of rats, and 28 differential metabolites were identified (Figure 2E and Supplementary Figure S3B and Table S2). A strong reduction of amino acids, such as phenylalanine, tyrosine, and leucine, was observed in DOX-treated rats compared to the control (Figure 2F). The levels of other metabolites, including lactic acid and N-acetylneuraminic acid, were higher in the DOX-treated group. Pathways involving amino acid metabolism were markedly altered under AIH, as indicated by the data (Figure 2G). Overall, the study on mouse and rat models provides insight into the metabolic alterations occurring in the liver due to AIH.

### 2.3. Network-Based Discovery of AIH-Associated Metabolic Signatures

To uncover actionable biomarkers for the characterization of AIH, we constructed a metabolite-metabolite network for each animal model. Spearman’s rank correlation coefficient (SCC) values of all pairs of differential metabolites between CON and DOX groups within a mouse or rat model were calculated. The top K strategy that used the K connections with the highest |SCC| was adopted to build the network. We tested cutoff values in the range from 10 to 80 with a step of five and identified the cutoffs of 35 for the mouse model and 20 for the rat model as producing the lowest network density (Supplementary Figure S4). Therefore, the top 30 connections were selected, and the highest *p*-value among all selected correlations was 0.021 (Supplementary Figure S5). To identify the metabolites with a strong influence on the network, we calculated the eigenvector centrality (EC) score for each. A high EC score indicates that a node is connected to many nodes that themselves have high scores [20].

**Figure 3:**
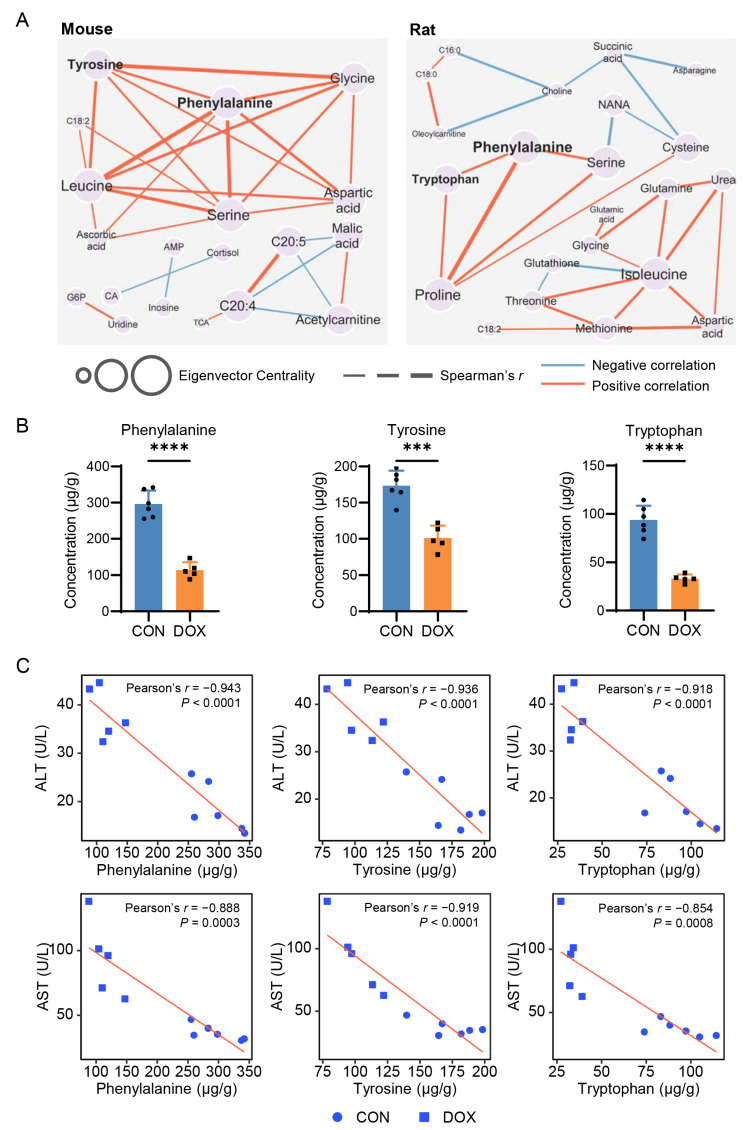
Network-based identification of potential biomarkers for anthracycline-induced hepatotoxicity (AIH). (**A**) Differential metabolite networks of AIH mouse and rat models. AMP: Adenosine monophosphate; CA: Cholic acid; G6P: Glucose 6-phosphate; TCA: Taurocholic acid. (**B**) Levels of aromatic amino acids (AAAs) in rat livers (CON, *n* = 6; DOX, *n* = 5). Student-*t* test, *** *p* < 0.001; **** *p* < 0.0001. (**C**) Correlations between hepatic AAAs and serum aminotransferases.

Five amino acids showed more robust connectivity than other metabolites in the mouse model network, including serine, leucine, phenylalanine, tyrosine, and glycine (Figure 3A). Phenylalanine and tyrosine are aromatic amino acids (AAA), and the conversion of phenylalanine to tyrosine in the liver is the first irreversible step in phenylalanine catabolism [21]. Interestingly, we observed that phenylalanine and tryptophan (which is also an AAA) also have strong connectivity and centrality in the network of the rat model (Figure 3A). Next, we inspected the levels of three AAAs in the DOX-treated rat livers using the LC-MS/MS-based targeted analysis. We found that hepatic phenylalanine, tyrosine, and tryptophan were significantly decreased (fold change [FC] < 0.6, *p* < 0.001) after DOX exposure (Figure 3B), consistent with the untargeted metabolomics analysis. Since serum ALT and AST are well-established biomarkers for clinical assessment of liver injury, we examined the correlation between three AAAs and two aminotransferases. As shown in Figure 3C, all three AAAs were significantly correlated with ALT (Pearson’s *r* = −0.91, *p* < 0.0001) and AST (Pearson’s *r* = −0.85, *p* < 0.001). These data highlight the importance of AAAs in understanding the pathogenesis of AIH and suggest their potential as biomarkers for characterizing this disease.

### 2.4. Doxorubicin Reduces Hepatic Phenylalanine and Tyrosine Levels in Breast Cancer Mice

**Figure 4:**
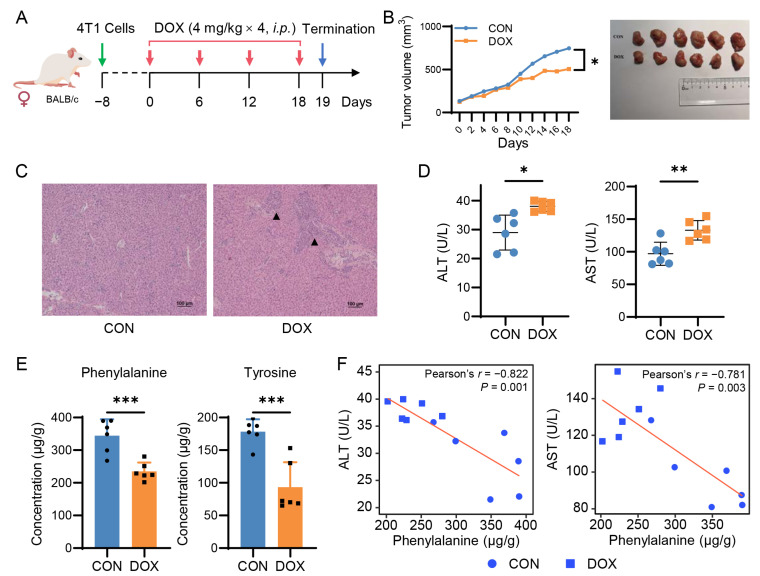
Doxorubicin reduces hepatic phenylalanine and tyrosine levels in breast cancer mice. (**A**) Schematic of a 4T1 tumor-bearing mouse model treated with doxorubicin (DOX). (**B**) DOX treatment delays tumor growth (*n* = 6 per group). Student-t test, * *p* < 0.05; ** *p* < 0.01; *** *p* < 0.001. (**C**) H&E staining of liver tissues. (**D**) Serum levels of ALT and AST. (**E**) Levels of phenylalanine and tyrosine in 4T1 tumor-bearing mouse livers. (**F**) Correlations between hepatic phenylalanine and serum aminotransferases.

To further confirm the significance of the network-discovered metabolites, we treated 4T1 breast cancer mice with DOX (Figure 4A). As expected, the administration of DOX delayed tumor growth (Figure 4B) and caused liver injury similar to that seen in mouse and rat models of AIH. The liver injury was characterized by pathological changes (Figure 4C) and elevated serum ALT and AST (Figure 4D). There was no change observed in cardiac function. Notably, targeted analysis using LC-MS/MS revealed a significant reduction in hepatic phenylalanine and tyrosine (FC < 0.7; *p* < 0.001), but not tryptophan, in the DOX-treated breast cancer mice, compared to the controls (Figure 4E and Figure S6A). In addition, we found a strong negative correlation with serum transaminases for phenylalanine (Pearson’s *r* = −0.78; *p* < 0.003), moderate for tyrosine (Pearson’s *r* = −0.64, *p* < 0.03), and weaker for tryptophan (Pearson’s r = −0.19; *p* = 0.557; Figure 4F and Figure S6B). These results suggest a consistent pattern of decreased phenylalanine and tyrosine levels in the liver after DOX exposure, which could have potential implications for AIH. However, additional experimental and clinical validation is necessary to confirm these findings.

## 3. Discussion

The hepatoxicity of anthracyclines is becoming a major concern in clinical anticancer practices, despite cardiotoxicity being the primary limitation associated with these drugs. We aimed to identify specific biomarkers of AIH by generating three animal models of low-dose DOX-induced hepatoxicity without cardiac dysfunction. We initially investigated the association between hepatic small molecule metabolites and DOX exposure in normal mouse and rat models using a mass spectrometry-based untargeted metabolomics approach. Consistent with previously reported studies [22,23], our metabolomic data revealed a variety of altered metabolites, including amino acids, nucleic acids, and lipids (Figure 1 and Figure 2). Notably, both essential and nonessential amino acids, such as serine, phenylalanine, and leucine, showed decreased levels after DOX treatment. One possible explanation is that DOX exposure could disrupt protein metabolism in the liver, leading to a decrease in amino acid levels. Additionally, we observed an accumulation of intermediates in glycolysis and TCA cycle pathways, such as glucose 6-phosphate, malic acid and succinic acid), along with a decrease in long-chain fatty acids (e.g., C18:2, C20:4, and C20:5) in the DOX group. These findings suggest that the development of AIH may be closely related to the inhibition of energy metabolism in liver cells. In fact, previous studies have demonstrated that DOX can interfere with mitochondrial function, resulting in decreased energy production [24].

To identify potential biomarkers from tens of differential metabolites, we then utilized advanced network science technology. The network method focuses more on connections between nodes and how those connections and nodes together combine into the whole system and less on precisely calculated values of each node statistics [25]. The network-based evaluation identified AAAs as candidate markers associated with AIH (Figure 3A). These network-predicted metabolic markers were highly correlated with the well-established biomarkers of liver damage (ALT and AST). Importantly, we confirmed the reduction in hepatic phenylalanine and tyrosine levels in the DOX-treated breast cancer mouse model through the use of LC-MS/MS targeted analysis.

Phenylalanine is an essential aromatic amino acid that acts as a growth and nutrient signal, regulates protein synthesis and degradation, and affects antidepressant and insulin secretion [26]. It is biologically converted into tyrosine, which is further converted into catecholamines, including dopamine, norepinephrine, and epinephrine. Several clinical studies have shown a significantly low level of phenylalanine in patients with alcoholic hepatitis and a low level of tyrosine in patients with non-alcoholic fatty liver disease (NAFLD) [27,28]. Sano et al. investigated the effects of a tyrosine-deficient diet in mice and showed that tyrosine deficiency could induce hepatic steatosis by disturbing the very low-density lipoproteins assembling through the Keap1-Nrf2 system [29]. Although emerging evidence suggests that AAA metabolism dysfunction is a major hallmark of diseased livers, the role of hepatic phenylalanine and tyrosine in developing AIH is still unclear. Our study provides the first compelling evidence for a reduction in phenylalanine and tyrosine levels in AIH.

The increased metabolic turnover is a possible reason for the decreased levels of hepatic phenylalanine and tyrosine under AIH. To test this option, we collected two RNA-seq datasets from the GEO database (https://www.ncbi.nlm.nih.gov/geo/, 4 May 2023) containing the gene expressions of rat primary hepatocytes treated with DOX. We examined the expression levels of the key genes involved in AAA catabolism and found that they were not significantly increased after DOX treatment (Supplementary Table S3). Therefore, we hypothesized that low levels of phenylalanine and tyrosine observed in AIH could be due to maldigestion caused by deficient digestive peptidase activity or liver dysfunction, which are common side effects of DOX [30].

Regardless of the exact mechanism, the observed reduction in hepatic phenylalanine and tyrosine levels could have important implications for cancer treatment, particularly for breast cancer patients who have a high incidence of AIH [9]. On the one hand, these two metabolites could potentially serve as biomarkers for predicting the development of AIH. On the other hand, restoring hepatic phenylalanine and tyrosine levels may contribute to alleviating AIH. Therefore, monitoring phenylalanine and tyrosine levels may provide valuable information for the management of cancer patients receiving anthracycline chemotherapy. However, these findings are modest, and more investigations are needed to validate whether phenylalanine and tyrosine are clinically useful biomarkers or therapeutic targets of AIH.

We acknowledge several potential limitations in the current study. We focused on AAAs because they ranked highly in the networks of both mouse and rat models. However, some other metabolites, such as leucine, serine, and arachidonic acid (C20:4), also have a strong influence over other nodes in the networks, providing potential candidates for AIH in further investigations. Integrating transcriptomics or proteomics data may help to better understand the complex association between AIH and metabolites. Additionally, obtaining liver biopsy samples from patients can be challenging and invasive. Thus, analyzing blood samples taken from patients treated with DOX is warranted to verify the potential of the two amino acids as blood markers for AIH. It is important to note that while the use of DOX alone may provide promising results in our study, it may not accurately represent the development of AIH in a clinical setting where patients are typically exposed to multiple drugs.

## 4. Materials and Methods

### 4.1. Chemicals and Reagents

Doxorubicin hydrochloride injection was bought from Shenzhen Main Luck Pharmaceuticals Inc. (Shenzhen, China). Derivatization reagents, including O-Methoxyamine hydrochloride (MOX·HCl) and N-Methyl-N-(trimethylsilyl)-trifluoroacetamide (MSTFA), were purchased from Sigma-Aldrich (St. Louis, MO, USA). Metabolite standards, such as phenylalanine, tyrosine, heptadecanoic acid, and 5,5,5-d_3_-Leucine, were also bought from Sigma-Aldrich (St. Louis, MO, USA). LC-MS grade acetonitrile, methanol, and ethyl acetate were purchased from Merck (Darmstadt, Germany) and formic acid was obtained from ROE Scientific, Inc. (Newark, NJ, USA). Ultrapure water was prepared using a Milli-Q system (Millipore, Bedford, MA, USA).

### 4.2. Cell Culture

Mouse mammary breast cancer 4T1 cells were purchased from the American Type Culture Collection (ATCC; Manassas, VA, USA) and cultured in RPMI-1640 medium media supplemented with a 10% fetal bovine serum (FBS) and 1% penicillin-streptomycin (Gibco, Grand Island, NY, USA) in a 37 °C incubator containing 5% CO_2_ with humidification.

### 4.3. Animal Experiments

Three animal models were used in this study, including DOX-treated mouse, rat, and 4T1 tumor-bearing mouse models. All animals were maintained in cages under standard laboratory conditions (12-h light/dark cycle) with free access to food and water.

*DOX-treated mouse model.* C57BL/6J male mice (7–8 weeks old, 20 ± 2 g) were purchased from the Jiangsu Huachuang Sino Pharma Tech Co., Ltd. (Taizhou, China). Animals were randomly divided into the CON (*n* = 6) and DOX (*n* = 8) groups. DOX at a dose of 3.0 mg/kg or sterile saline was administered i.p. every other day for two weeks (a total of eight times). Finally, mice were analyzed by echocardiography and then sacrificed under anesthesia 2 weeks after the last injection.

*DOX-treated rat model.* Sprague–Dawley male rats (7–8 weeks old, 200 ± 20 g) were purchased from Sino-British SIPPR/BK Lab Animal Ltd. (Shanghai, China). Animals were randomly divided into control (CON, *n* = 6) and DOX (*n* = 8) groups. DOX at a dose of 2.5 mg/kg or sterile saline was administered intraperitoneally (i.p.) every 2 days for 6 consecutive times. Finally, the rats were analyzed by echocardiography and then sacrificed under anesthesia five days after the last injection.

*DOX-treated breast cancer mouse model.* BALB/c female mice (6–7 weeks old, 20 ± 2 g) were purchased from Changzhou Cavins Laboratory Animal Co., Ltd. (Changzhou, China). 4T1 breast cancer cells, suspended at 2 × 10^5^ cells in 50 μL of medium mixed with 50 μL of matrigel basement membrane matrix, were injected into the fourth pair of mammary fat pads of mice under isoflurane anesthesia. Tumor diameters were recorded every two days and calculated using the following formula: tumor volume = 1/2 × length × width × height. Once the tumor volumes reached 50–100 mm^3^, mice were randomly divided into CON (*n* = 6) and DOX (*n* = 6) groups. DOX at a dose of 4.0 mg/kg or sterile saline was administered i.p. every 6 days for 4 consecutive times. Finally, mice were analyzed by echocardiography and then sacrificed under anesthesia the day after the last injection.

For each animal experiment, we randomly selected three animals from each group and fixed their right liver lobes slices with formalin for histopathological examination. Other liver tissues and serum samples were collected and stored at −80 °C for further analysis. All animal procedures complied with the Guide for the Care and Use of Laboratory Animals and were approved by the Animal Ethics Committee of China Pharmaceutical University.

### 4.4. Histopathological and Biochemical Analysis

The liver samples were fixed in a 4% Paraformaldehyde Fix Solution and embedded in paraffin. Sections were cut at 2 μm from the paraffin blocks and stained with hematoxylin and eosin (H&E) according to standard procedures. Serum levels of ALT and aspartate transaminase (AST) were measured by microplate method using commercial assay kits (Nanjing Jiancheng Bioengineering Institute, Nanjing, China).

### 4.5. LC-MS and GC-MS Based Untargeted Metabolomics Analysis

Liver samples were homogenized with an ice-cold homogenization solution (methanol/water = 80/20, *v*/*v*) at a ratio of 1:10 (*w*/*v*, mg/μL). The mixture was centrifuged for 10 min at 6000× *g* at 4 °C. The supernatant was transferred and divided into 2 parts: one (40 μL) for LC-MS analysis and the other one (10 μL) for GC-MS analysis. In addition, to ensure the reliability of the analytical process, pooled quality control (QC) samples were prepared by mixing equal aliquots (50 μL) of liver homogenates.

For LC-MS analysis, the liver homogenate was added with 40 µL of pre-cold methanol containing glibenclamide (5 µg/mL, internal standard), vortexed for 3 min, and twice centrifuged (19,000× *g*, 4 °C, 10 min). A 5-μL aliquot of the supernatant was injected into a Shimadzu ultrafast liquid chromatography ion trap/time-of-flight mass spectrometry system (UFLC-IT-TOF/MS, Shimadzu, Tokyo, Japan). Samples were separated on a Waters XSelect HSS T3 XP column (2.1 × 100 mm, 2.5 μm, Waters, Milford, MA, USA) with programmed gradient elution. MS data were acquired between m/z 70 and 1000 using an electrospray ionization (ESI) source operated under positive and negative switching modes. More detailed parameters are shown in the Supplementary Materials.

For GC-MS analysis, the liver homogenate was added to 100 µL of pre-cold methanol containing heptadecanoic acid (5 µg/mL, internal standard) and vortexed for 3 min. After centrifugation (19,000× *g*, 4 °C, 10 min), 80 µL of supernatant was transferred to a clean glass vial and subsequently processed by a two-step derivatization method using MOX (10 mg/mL) and MSTFA (20% in acetic ether). After derivatization, a 1-μL aliquot of the mixture was injected into a Shimadzu GCMS-QP2010 system (Shimadzu, Tokyo, Japan) equipped with an SH-Rxi-5Sil MS column (30.0 m × 0.25 mm, 0.25 μm, Restek, Bellefonte, PA, USA). Other detailed conditions of GC-MS analysis are described in the Supplementary Materials.

### 4.6. Identification of Differential Metabolites

The raw data obtained from LC-MS and GC-MS analyses were imported to the Profiling Solution software for peak detection and alignment. Subsequently, the data matrix was processed using the muma, factoextra, and ropls packages in R, including missing value replacement, normalization, scaling, PCA, and OPLS-DA. The VIP value > 1 and FC > 1.2 or <1/1.2 were set as cutoffs to screen out the differential features. The commercial or online-free databases were used for the preliminary identification of metabolites: the NIST 11 mass spectral library for GC-MS data and the HMDB database for LC-MS data. Finally, the structure of some metabolites was confirmed using chemical standards.

### 4.7. LC-MS/MS Targeted Analysis

Determination of aromatic amino acids in liver tissues was performed on a Shimadzu LCMS-8040 system (Shimadzu, Tokyo, Japan). The separation was performed on an XBridge BEH Amide column (2.1 × 100 mm, 2.5 μm, Waters, Milford, MA, USA) column with a flow rate of 0.3 mL/min at 35 °C. The gradient elution involved a mobile phase consisting of (A) 5 mM ammonium acetate in water with formic acid adjusting pH to 3.0 and (B) acetonitrile. The MS data were acquired in the positive mode using multiple reaction monitoring (MRM). The additional information is given in the Supplementary Materials.

### 4.8. Network Analysis

We utilized Mathematica v13.0 (Wolfram Research, Champaign, IL, USA) to construct the metabolite-metabolite networks for rat and mouse models, respectively. The SCC values of all pairs of differential metabolites were calculated. The 30 connections with the highest SCC were used to build the network for each animal model. The networks were visualized using Cytoscape v3.9.1. For evaluation, EC was used to rank the metabolites (nodes) in the networks. We then checked the Person’s correlation between 2 serum aminotransferases (ALT and AST) and some metabolites that were important to the networks.

### 4.9. Statistical Analysis

Student’s *t*-test and a Pearson correlation analysis were performed on Mathematica software. *p* < 0.05 was considered statistically significant.

## 5. Conclusions

In summary, our study employed multiple approaches, including metabolomics and network analysis, to identify metabolic signatures of AIH in both normal and breast cancer animal models. We utilized untargeted metabolic profiling, targeted LC-MS/MS analysis, systems biology, and external validation to uncover reliable biomarkers of AIH. Our study highlighted the significant reduction in hepatic phenylalanine and tyrosine levels, which were highly correlated with serum ALT and AST levels across all three animal models following DOX exposure. These results provide crucial insights into the relationship between chemotherapy-induced liver injury and the current management of cancer treatment. We believe that our findings will bridge critical knowledge gaps by providing valuable information that can guide future research toward developing more effective interventions to manage AIH in cancer patients.

## Data Availability

The R and Mathematica codes for statistical analysis and network analysis are available on our lab’s Gitee website (https://gitee.com/huanglab/doxorubicin-hepatotoxicity-network). Any additional information required to reanalyze the data reported in this paper is available from the lead contact upon request.

## Supplementary Materials

The following supporting information can be downloaded at: https://www.mdpi.com/article/10.3390/ph16060797/s1, Figure S1: Results of the pilot experiment; Figure S2: Untargeted metabolomic analysis of AIH mouse livers; Figure S3: Untargeted metabolomic analysis of AIH rat livers; Figure S4: Network density-based K cutoff selection; Figure S5: Distribution of the *p*-values of selected connections; Figure S6: LC-MS/MS analysis on 4T1 tumor-bearing mice; Table S1: 27 differential metabolites identified from mouse model; Table S2: 28 differential metabolites identified from rat model; Table S3: the expression levels of key genes involved in AAA catabolism.
