# Supplementary material for: Metabolomics and Network Analyses Reveal Phenylalanine and Tyrosine as Signatures of Anthracycline-Induced Hepatotoxicity
Source: Pharmaceuticals (Basel). 2023 May 26;16(6):797. doi: 10.3390/ph16060797 (PMC10302024; doi:10.3390/ph16060797)
Supplement: Supplementary file 1 [file pharmaceuticals-16-00797-s001.zip › pharmaceuticals-2374324-supplementary.pdf]

Supplementary Information

Metabolomics and network analyses reveal phenylalanine and tyrosine as signatures of anthracycline-induced hepatotoxicity

Peipei Liu[1#], Jing Wu[2,3#], Xinyue Yu[2], Linling Guo[2,3], Ling Zhao[1], Tao Ban[1,4*], Yin Huang[2,3*]

[1] Department of Pharmacology, College of Pharmacy, Harbin Medical University, Harbin, China.

[2] Key Laboratory of Drug Quality Control and Pharmacovigilance, China Pharmaceutical University, Ministry of Education, Nanjing, 210009, China

[3] Department of Pharmaceutical Analysis, School of Pharmacy, China Pharmaceutical University, Nanjing, 210009, China

[4] Heilongjiang Academy of Medical Sciences, Harbin 150081, China

[#] These authors contributed equally to this work.

Document S1

LC-MS conditions

The sample was injected into a Shimadzu ultrafast liquid chromatography ion trap/time-of-flight mass spectrometry (UFLC-IT-TOF/MS, Shimadzu, Tokyo, Japan) system. Samples were separated on a Waters XSelect HSS T3 XP (2.1×100 mm, 2.5 μm, Waters, Milford, MA, USA) column at the oven temperature of 40°C. The mobile phase consisted of 0.1% (v/v) formic acid in water (A) and methanol (B) with a flow rate of 0.3 mL/min. A 28-min elution gradient was performed as follows: 0-6 min, 1% B; 6-7 min, 1%-35% B; 7-13 min, 35% B; 13-14 min, 35%-50% B; 14-16 min, 50% B; 16-17 min, 50%-75% B; 17-20 min, 75% B; 20-21 min, 75%-100% B; 21-25 min, 100% B; Finally, the initial conditions were recovered and kept for 9 min.

MS detection was performed by an electrospray ionization (ESI) source operated in both positive and negative modes with the data acquisition range of *m/z* 70-1000. The other main parameters were as follows: interface voltage 4.5 kV and -3.5 kV, curved desorption line temperature 200°C, heater block temperature 200°C, and ion accumulation time 20 ms.

GC-MS conditions

After derivatization, a 1-μL aliquot of the mixture was injected into a Shimadzu GCMS-QP2010 system (Shimadzu, Tokyo, Japan) equipped with an SH-Rxi-5Sil MS column (30.0 m × 0.25 mm, 0.25 μm, Restek, USA). Helium ran as carrier gas at a flow rate of 1.0 mL/min. A 32- minute temperature program was performed as follows: the oven temperature was set at 70°C during the first 2 min, then linearly changed to 320°C in 25 min, and kept for 2 min; finally, the initial conditions were recovered and maintained for 3 min.

MS detection was carried out using electron impact mode (70 eV) and full scan monitoring (*m/z* 45 to 600). The temperatures of injector and ion source were set at 250°C and 200°C, respectively.

LC-MS/MS conditions

The sample was injected into a Shimadzu LCMS-8040 system (Shimadzu, Tokyo, Japan). Chromatographic separation was achieved on a Waters XBridge BEH Amide Column (2.1×100 mm, 2.5 μm, Waters, Milford, MA, USA) equipped with an X Bridge BEH Amide 2.5 μm VanGuard precolumn, and the column temperature was maintained at 35°C. The mobile phase consisted of (A) 5 mM ammonium acetate in water with formic acid adjusting pH to 3.0 and (B) acetonitrile. The flow rate of the mobile phase was 0.3 mL/min. The program of gradient elution was as follows: 0-7 min, 85%-82% B; 7-9 min, 82-50% B; 9-11 min, 50% B; 11-12 min: 50-85% B.

Mass spectrometric detection was accomplished by an electrospray ionization (ESI) source operated in positive mode with multiple-reaction monitoring (MRM). The optimized parameters were as follows: spray voltage 4.0 kV, the desolvation line temperature 250°C, the heat block temperature 400°C, scan width for MRM 0.1m/z, nebulizing gas 3.0 L/min, drying gas 10.0 L/min. The specific MRM transitions employed for quantitation of Phe, Tyr, and Trp were *m/z* 166.1>120.1, 182.1>91.1, and 205.1> 88.0, respectively. The optimized collision energies of Phe, Tyr, and Trp were -14, -28, and -11 V, respectively.

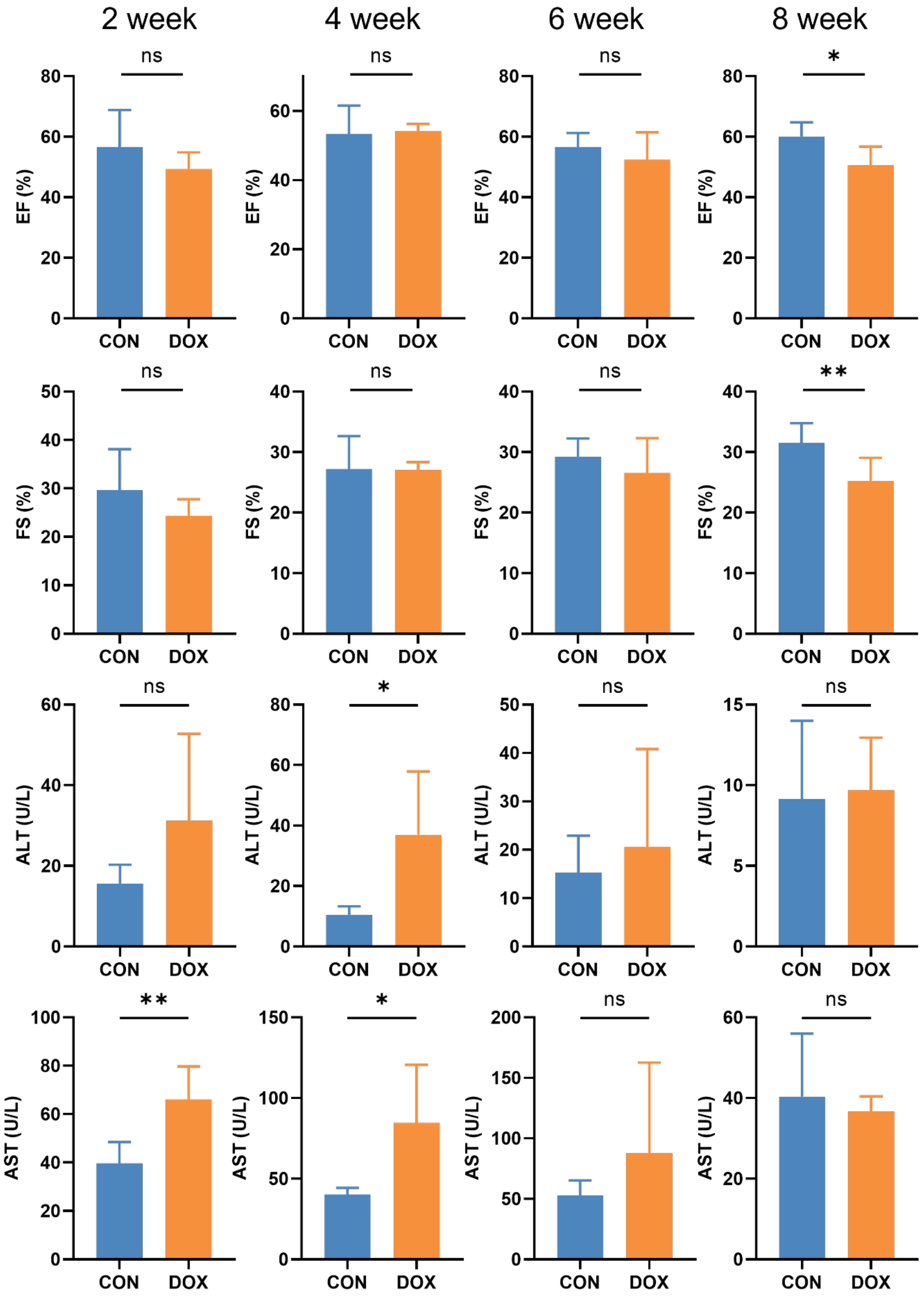

Fig. S1 The pilot experiment shows that mice develop the most severe liver injury at 4th week after DOX exposure, while heart function is unaffected. n = 6 per group. Student-t test, * P < 0.05; ** P < 0.01; ns, P > 0.05.

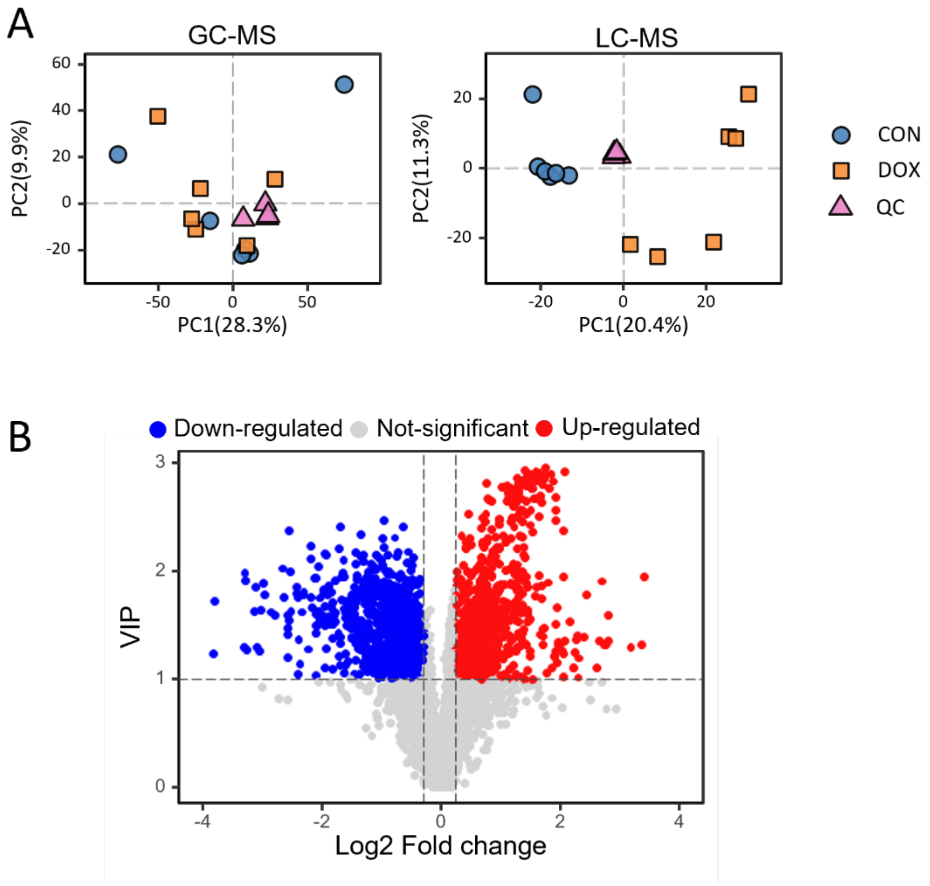

Fig. S2 Untargeted metabolomic analysis of AIH mouse livers. (A) Principal component analysis (PCA) for the data obtained from LC-MS and GC-MS analyses. (B) Volcano plot for all features.

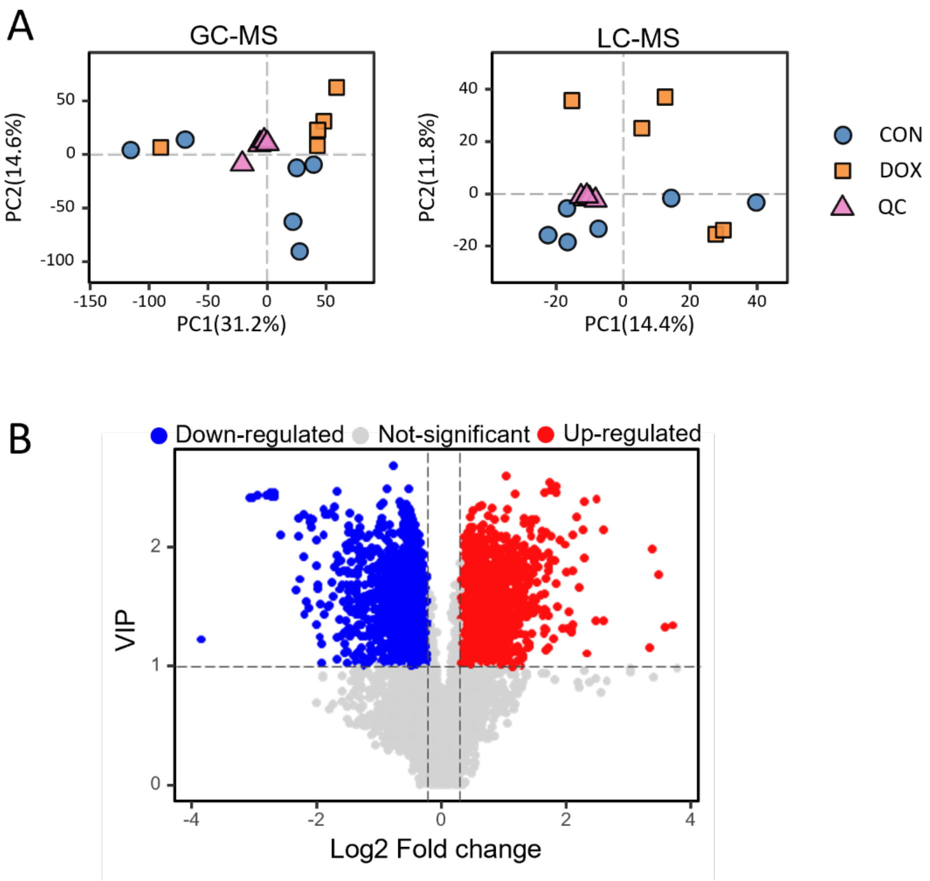

Fig. S3 Untargeted metabolomic analysis of AIH rat livers. (A) Principal component analysis (PCA) for the data obtained from LC-MS and GC-MS analyses. (B) Volcano plot for all features.

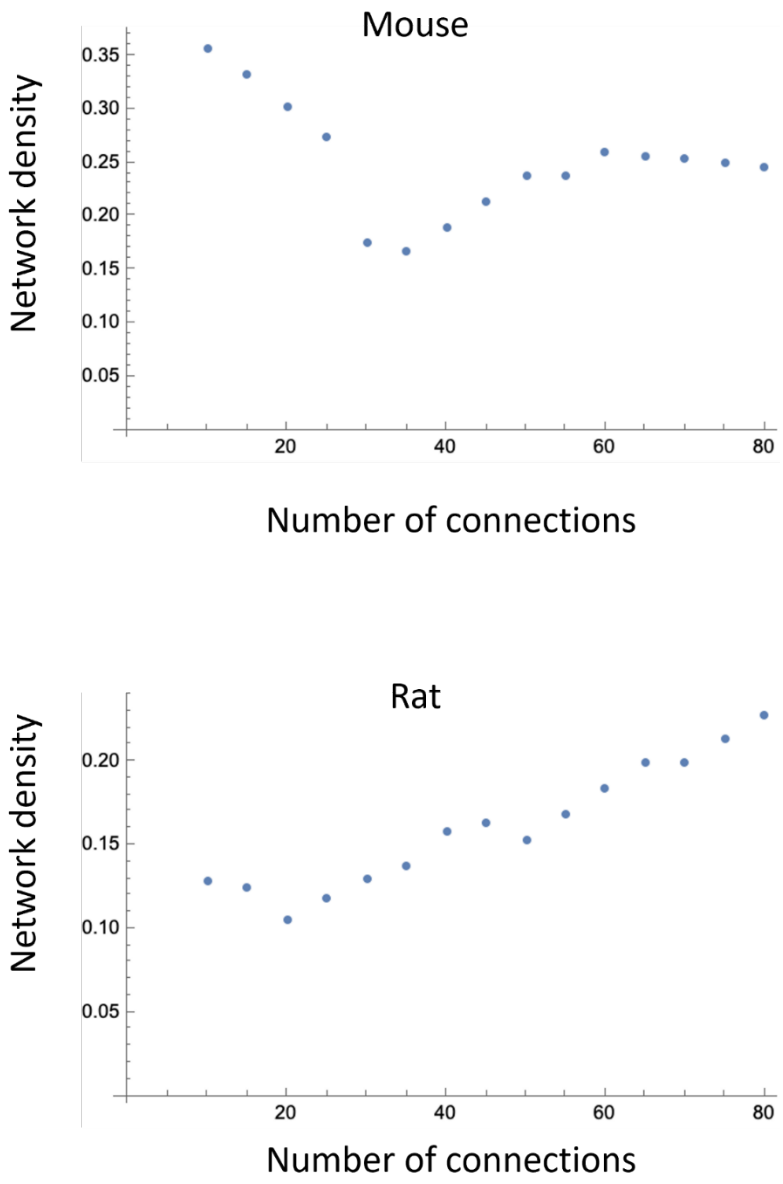

Fig. S4 Network density-based K cutoff selection.

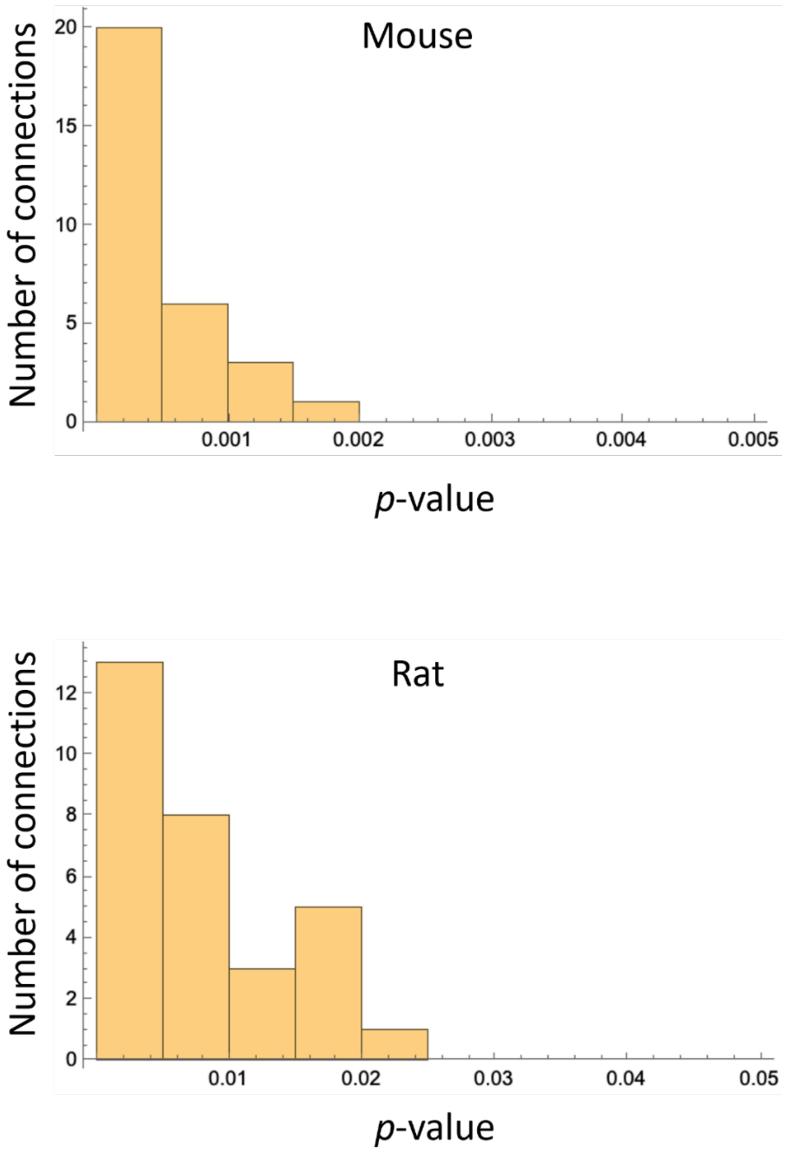

Fig. S5 Distribution of the *p*-values of selected connections.

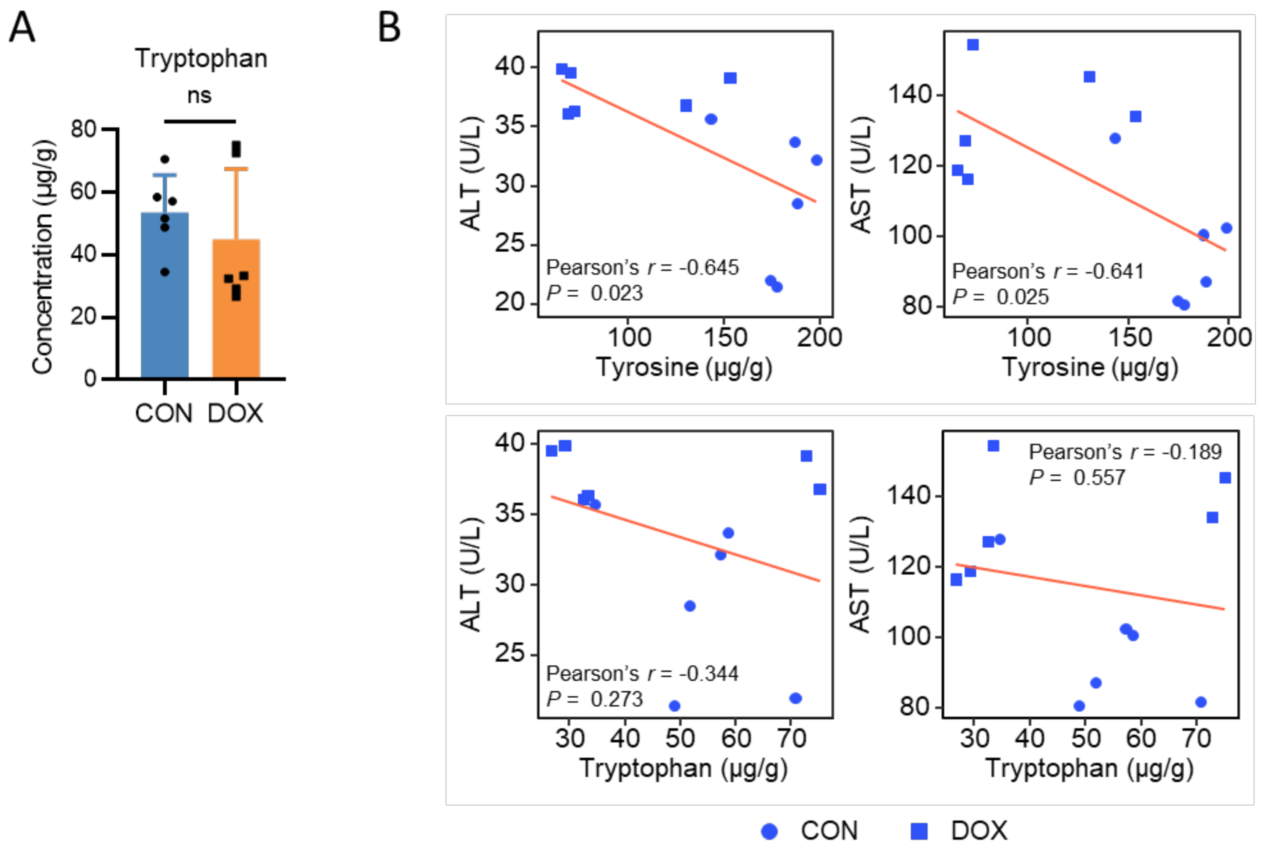

Fig. S6 (A) Hepatic tryptophan level in two groups of 4T1 tumor-bearing mice. (B) Correlations of hepatic tyrosine and tryptophan with serum ALT and AST. Student-t test, ns, $P > 0.05$.

Table S1 27 differential metabolites identified from mouse model.

LC-MS analysis

Metabolite	HMDB ID	Category	Measure RT (min)	Measured m/z	Adduct	VIP	FC	MSI level
Lysine*	HMDB0000182	Amino acids	0.744	147.1068	M+H	1.015	1.438	1
Glutamine*	HMDB0000641	Amino acids	0.916	147.0766	M+H	1.568	0.707	1
Acetylcarnitine*	HMDB0000201	Fatty acyls	0.949	204.1193	M+H	1.531	2.513	1
Malic acid*	HMDB0000156	Organic oxides	0.974	133.0179	M-H	1.302	1.473	1
Inosine*	HMDB0000195	Nucleosides and nucleotides	2.670	267.0736	M-H	1.468	1.249	1
Cortisol*	HMDB0000063	Organic oxides	8.983	361.2027	M-H	1.397	2.581	1
Taurodeoxycholic acid*	HMDB0000896	Bile acids	11.921	500.3037	M+H	1.092	0.693	1
Oleoylcarnitine*	HMDB0005065	Fatty acyls	12.138	426.3558	M+H	1.094	1.725	1
Taurocholic acid*	HMDB0000036	Bile acids	12.919	516.3000	M+H	1.114	0.565	1
C20:5*	HMDB0001999	Fatty acyls	14.163	301.2179	M-H	1.601	0.489	1
C20:4*	HMDB0001043	Fatty acyls	14.174	303.2236	M-H	1.288	0.385	1
Dihydroxyacetone phosphate	HMDB0001473	Organic oxides	0.974	168.9916	M-H	1.249	1.964	2
Glucose 6-phosphate	HMDB0001401	Organic oxides	0.982	259.0224	M-H	1.012	1.342	2
Adenosine monophosphate	HMDB0000045	Nucleosides and nucleotides	1.448	348.0684	M+H	2.121	0.223	2
Uridine	HMDB0000296	Nucleosides and nucleotides	1.864	243.0617	M-H	1.150	1.217	2
Deoxyguanosine	HMDB0000085	Nucleosides and nucleotides	3.188	268.1011	M+H	1.782	0.683	2
Cholic acid	HMDB0000619	Bile acids	10.745	407.2812	M-H	1.261	0.495	2

GC-MS analysis

Metabolite	HMDB ID	Category	MW	Measure RT (min)	VIP	FC	MSI levels
Glycine*	HMDB0000123	Amino acids	75.067	7.372	1.056	0.667	1
Leucine*	HMDB0000687	Amino acids	131.173	9.845	1.132	0.633	1
Succinic acid*	HMDB0000254	Organic oxides	118.088	10.315	1.635	2.124	1
Serine*	HMDB0000187	Amino acids	105.093	11.165	1.050	0.682	1
Aspartic acid*	HMDB0000191	Amino acids	133.103	13.399	1.200	0.563	1
Glutamic acid*	HMDB0000148	Amino acids	147.129	14.684	1.081	0.815	1
Phenylalanine*	HMDB0000159	Amino acids	165.189	14.838	1.067	0.678	1
Tyrosine*	HMDB0000158	Amino acids	181.189	18.403	1.030	0.698	1
C18:2*	HMDB0006270	Fatty acyls	280.446	21.165	1.142	0.649	1
Ascorbic acid	HMDB0000044	Organic oxides	176.124	18.463	1.065	0.736	2

* Confirmed by chemical standards. FC: fold change (DOX/CON).

Table S2 28 differential metabolites identified from rat model.

LC-MS analysis

Metabolite	HMDB ID	Category	Measure RT (min)	Measured m/z	Adduct	VIP	FC	MSI level
Taurine*	HMDB0000251	Organic oxides	0.875	124.0141	M-H	1.491	0.539	1
Proline*	HMDB0000162	Amino acids	0.953	116.0712	M+H	1.569	0.561	1
N-Acetylneuraminic acid*	HMDB0000230	Organic oxides	0.958	308.0945	M-H	1.945	1.717	1
Tyrosine*	HMDB0000158	Amino acids	1.647	180.0657	M-H	1.527	0.725	1
Phenylalanine*	HMDB0000159	Amino acids	3.303	166.0854	M+H	1.811	0.664	1
Tryptophan*	HMDB0000929	Amino acids	4.278	203.0823	M-H	1.129	0.714	1
Oleoylcarnitine*	HMDB0005065	Fatty acyls	12.273	426.3570	M+H	1.100	1.912	1
C20:5*	HMDB0001999	Fatty acyls	14.393	301.2118	M-H	1.341	0.550	1
Choline	HMDB0000097	Cholines	0.853	104.1064	M+H	1.236	0.675	2
Glutathione	HMDB0000125	Organic oxides	1.351	308.0867	M+H	1.243	1.823	2

GC-MS analysis

Metabolite	HMDB ID	Category	MW	Measure RT (min)	VIP	FC	MSI level
Glycine*	HMDB0000123	Amino acids	75.067	7.242	1.467	0.784	1
Leucine*	HMDB0000687	Amino acids	131.173	7.869	1.380	0.587	1
Proline*	HMDB0000162	Amino acids	115.131	8.184	1.993	0.642	1
Isoleucine*	HMDB0000172	Amino acids	131.173	10.032	1.934	0.768	1
Threonine*	HMDB0000167	Amino acids	119.119	10.039	2.151	0.673	1
Succinic acid*	HMDB0000254	Organic oxides	118.088	10.312	1.400	1.228	1
Serine*	HMDB0000187	Amino acids	105.093	10.992	2.174	0.675	1
Methionine*	HMDB0000696	Amino acids	149.211	11.778	1.685	0.789	1
Aspartic acid*	HMDB0000191	Amino acids	133.103	11.919	1.253	0.791	1
Glutamine*	HMDB0000641	Amino acids	146.145	13.246	1.369	0.817	1
Cysteine*	HMDB0000574	Amino acids	121.158	13.674	1.275	0.819	1
Glutamic acid*	HMDB0000148	Amino acids	147.129	14.472	1.017	0.795	1
Phenylalanine*	HMDB0000159	Amino acids	165.189	14.604	1.693	0.765	1
Asparagine*	HMDB0000168	Amino acids	132.118	15.074	1.057	0.810	1
C16:0*	HMDB0000220	Fatty acyls	256.424	19.271	1.177	1.327	1
C18:2*	HMDB0006270	Fatty acyls	280.446	20.934	1.085	0.408	1
C18:0*	HMDB0000827	Fatty acyls	284.477	21.169	1.013	1.291	1
C20:4*	HMDB0001043	Fatty acyls	304.467	21.811	1.200	0.659	1
Lactic acid	HMDB0000190	Organic oxides	90.078	8.269	1.171	1.264	2
Urea	HMDB0000294	Organic oxides	60.055	9.227	1.208	0.793	2

* Confirmed by chemical standards. FC: fold change (DOX/CON).

Table S3 The expression levels of key genes involved in AAA catabolism.

GEO available	Sample	Gene name	adj.P.Val	P.Value	logFC
GSE59906	Rat Primary Hepatocytes	*Ddc*	0.483	0.119	0.487
GSE59906	Rat Primary Hepatocytes	*Got2*	0.568	0.188	-0.456
GSE59906	Rat Primary Hepatocytes	*Aoc3*	0.702	0.343	0.410
GSE59906	Rat Primary Hepatocytes	*Pah*	0.315	0.039	-0.358
GSE59906	Rat Primary Hepatocytes	*Tat*	0.453	0.099	-0.317
GSE59906	Rat Primary Hepatocytes	*Aldh3a1*	0.819	0.526	0.156
GSE59906	Rat Primary Hepatocytes	*Aldh3a2*	0.785	0.473	-0.124
GSE59906	Rat Primary Hepatocytes	*Maob*	0.966	0.875	0.049
GSE152128	Rat Primary Hepatocytes	*Tat*	0.000	0.000	-1.143
GSE152128	Rat Primary Hepatocytes	*Aldh3a1*	0.000	0.000	3.458
GSE152128	Rat Primary Hepatocytes	*Maoa*	0.360	0.233	0.093
GSE152128	Rat Primary Hepatocytes	*Aldh3b1*	0.940	0.908	0.034
GSE152128	Rat Primary Hepatocytes	*Tyro3*	0.011	0.002	0.602
